# Supplementary material for: A quality management system aiming to ensure regulatory-grade data quality in a glaucoma registry
Source: PLoS One. 2023 Jun 2;18(6):e0286669. doi: 10.1371/journal.pone.0286669 (PMC10237471; doi:10.1371/journal.pone.0286669)
Supplement: S1 Table — (DOCX) [file pone.0286669.s001.docx]

**S1 Table. Contents of functional tests and user acceptance tests**

1. **Functional tests**

| **Test ID** | **Purpose of test** |
| --- | --- |
| FN001 | Verify the setting of “Role and privilege” as expected. |
| FN002 | Verify the setting of “General” as expected. |
| FN003 | Verify the setting of “Research site” as expected. |
| FN004 | Verify the setting of “Group” as expected. |
| FN005 | Verify the setting of “Visits” as expected. |
| FN006 | Verify the setting of “Form” as expected. |
| FN007 | Verify the setting of “Audit” as expected. |
| FN008 | Verify the setting of “Skip logic” as expected. |
| FN009 | Verify the setting of “Query” as expected. |
| FN010 | Verify the setting of “Form rule” as expected. |
| FN011 | Verify the setting of “Subject status rule” as expected. |

1. **User acceptance tests (UATs)**

| **Test ID** | **Purpose of test** |
| --- | --- |
| RQ001 | Verify the issuing and activating of a Medrio user account for the registry |
| RQ002 | After entering the subject's eligibility, confirm that the subject identification number is issued and displayed. If subject is not eligible, ensure that any further registration does not proceed. |
| RQ003 | Verify all data and inputs. Use test data that covers the maximum number of fields, and confirm that all data can be entered, including forms and fields that appear depending on the input. |
| RQ004 | Verify behavior when an unscheduled visit is added. |
| RQ005 | Confirm that the status is changed according to the input settings of each form. |
| RQ006 | Verify that query display, query resolution, and approval can be performed according to the assumed procedure. Verify that the query cannot be approved unless it is resolved. |
| RQ007 | Verify data processing (soft lock, SDV, data review) operations with DM, CRA privileges. Ensure that re-signing is required for post-signing modifications. |
| RQ008 | After performing the steps of RQ003, RQ006, RQ007 on the subject of the target test scenario, fix the data and confirm that the data cannot be subsequently changed. |
| RQ009 | If it becomes necessary to correct the data after fixing the data, release the data fix with DM, correct it, and then perform SDV, signature, and review again to verify that the data can be fixed again. |
| RQ010 | The input value should be output normally as PDF, CSV, and SAS. Also, ensure that the data in the other analysis tabs is exported. Visually confirm that each subject and visit can be easily identified in the data set. |
| RQ011 | Verify that the progress can be confirmed using the report of the input data. |
| RQ012 | Check the operation of the Project Manager and Read Only accounts, which are Read Only functions. |

Abbreviations: CRA, clinical research associate; CSV, computerized system validation; DM, data manager; PDF, Portable Document Format; SAS, Statistical Analysis System; SDV, source data verification
