# Supplementary material for: A quality management system aiming to ensure regulatory-grade data quality in a glaucoma registry
Source: PLoS One. 2023 Jun 2;18(6):e0286669. doi: 10.1371/journal.pone.0286669 (PMC10237471; doi:10.1371/journal.pone.0286669)
Supplement: S2 Table — (DOCX) [file pone.0286669.s002.docx]

**S2 Table. Assessment of addressed recommendations related to data quality in the regulatory guidelines**

| **FDA- Use of Real-world Evidence to Support Regulatory Decision-making for Medical Devices** | | | |
| --- | --- | --- | --- |
| **No.** | **Recommendation in the guidance** | **Does the Glaucoma registry meet the recommendation?** | |
| 1 | 1) Data Accrual [1] The preparedness of individual sites for complete and accurate collection of RWD (e.g., whether there are defined processes, site training and support, and qualified personnel) | Yes | The process of data entry from source documents to CRF is standardized by the EDC operating manual and CRF data entry manual. Data entry users underwent the first EDC user training concerning these manuals at the start-up meeting and were granted training certification before starting the process of data entry. Ad-hoc user training is performed as manuals are revised and new study staff joins. |
| 2 | [2] Whether a common data capture form was used | Yes | Data collection formats and structures are configurated in accordance with CDASH and the common entry form equipped in Medrio EDC, except for examinations and diagnostic images specific for ophthalmology. The definitions of the data elements and fields for ophthalmology tests are configurated in reference to existing clinical trials of glaucoma. Data capture is conducted in the EDC system. |
| 3 | [3] Whether a common definitional framework (i.e., data dictionary) was used | No | A data coding dictionary (e.g. MedDRA and WHO drug) has not been incorporated in the EDC system for the glaucoma registry yet. |
| 4 | [4] Adherence to a common temporal framework for collection of key data points | Yes | The timing of data collection with visit window is pre-specified in the protocol. |
| 5 | [5] The timing of establishing the study plan, protocol, and/or analysis plan relative to collection or retrieval of the RWD | Yes | The registry protocol, including study plan and data collection, is finalized prior to data collection. An analysis plan is to be developed separately. |
| 6 | [6] The sources and technical methods used for data element capture (e.g., chart abstraction, point of care entry, EHR integration, UDI capture, data records from the device, and linkage to claims data) | Yes | The source, type, format and method of data generation from the registry is pre-defined in the data flow diagram before starting data collection. |
| 7 | [7] Whether patient selection and enrollment criteria minimize bias and ensure a representative real-world population (e.g., all-comer’s design, consecutive patient enrollment) | Yes | Inclusion and exclusion criteria for the registry are pre-specified in the protocol and minimal exclusion criteria are set for the glaucoma registry to allow consecutive patient enrollment in the registry. |
| 8 | [8] The timeliness of data entry, transmission, and availability | Yes | Due date of data entry is pre-determined in the data entry manual. The timeliness of data entry is ensured by central monitoring as stipulated in the monitoring manual. |
| 9 | [9] Whether necessary and adequate patient protections were in place (e.g., methods to protect patient privacy, and need for informed consent as determined by the reviewing IRB and in compliance with FDA regulations). | Yes | The protocol of the registry was reviewed and approved by an ethical review board, and stipulates the process of informed consent from participants. Handling of personal information and anonymization of personal information are specified in the registry protocol. |
| 10 | 2) Data assurance – quality control [1] The quality of the data element population (e.g., whether abstracted from a verifiable source to assess transcription errors or automatically populated through a data extraction algorithm) | Yes | Data transcription and entry of study data from verifiable source documents, including the EHR to EDC system, is operated manually by designated trained users. |
| 11 | [2] Adherence to source verification procedures and data collection and recording procedures for completeness and consistency | Yes | Data collection and recording procedures in the EHR are consistent with these processes in clinical practice. The data entry process is pre-specified in the EDC operating manual and CRF entry manual. The monitoring plan pre-specified regular on-site monitoring by a designated site monitor at least every 3 months to perform SDV for all key data fields. Remote monitoring by data manager was planned to monitor compliance to the protocol through the EDC system at the frequency of at least every 3 months. |
| 12 | [3] Completeness (i.e., minimized missing or out of range values) of data necessary for specified analyses, including adjustment for confounding factors | Yes | The monitoring plan pre-specified regular on-site monitoring by a designated site monitor at least every 3 months to perform SDV for all key data fields. Remote monitoring by data manager was planned to monitor compliance to the protocol through the EDC system at the frequency of at least every 3 months. Missing or out of range values are checked automatically in the EDC system and reviewed in on-site and remote (central) monitoring at the level predefined by the manuals. All relevant queries should be addressed. Ensuring that there are no missing data in the critical data is predefined in the monitoring plan. |
| 13 | [4] Data consistency across sites and over time | Yes | Data consistency across sites and over time is ensured by use of a standardized data input format used in the EDC system and controlled by the EDC operating manual and CRF data entry manual. |
| 14 | [5] Evaluation of on-going training programs for data collection and use of data dictionaries at participating sites | Yes | The process of data entry from source documents to CRF is standardized in the EDC operating manual and CRF data entry manual. Data entry users underwent the first EDC user training for these manuals at the start-up meeting and were granted a training certificate before starting the process of data entry. Ad-hoc user training is performed as manuals are revised and new study staff joins. |
| 15 | [6] Evaluation of site and data monitoring practices | Yes | Regular audit is planned to ensure the quality of the glaucoma registry. |
| 16 | [7] The use of data quality audit programs. | Yes | Regular audit is planned to ensure the quality of the glaucoma registry. |

| **EMA- Discussion paper: Use of patient disease registries for regulatory purposes - methodological and operational considerations** | | | |
| --- | --- | --- | --- |
| **No.** | **Recommendation in the guidance** | **Does the Glaucoma registry meet the recommendation?** | |
| 1 | 1) Patient population: Great care should be exercised to ensure exhaustive enrolment of patients and avoid selection bias | Yes | Inclusion and exclusion criteria for the registry are pre-specified in the protocol and minimal exclusion criteria are set for the glaucoma registry to allow consecutive patient enrollment in the registry. |
| 2 | 2) Time elements: Accurate knowledge and recording of dates of important events | Yes | Dates of important events (e.g. site visits, glaucoma diagnosis, start or end of treatment) are captured in the EDC system and reviewed in on-site monitoring. |
| 3 | 3) Core data elements:  Core data elements and formats should be harmonized or mapped across registries for the same disease | Yes | Data collection formats and structures are configurated in accordance with CDASH and the common entry form equipped in Medrio EDC, except for examinations and diagnostic images specific for ophthalmology. The definitions of the data elements and fields for ophthalmology tests are configurated in reference to existing clinical trials of glaucoma. Data capture is conducted in the EDC system. |
| 4 | 4) Terminologies:  As part of harmonization between registries covering the same disease, common terminologies should be used across registries for diseases, diagnostic tests, symptoms, medicinal products, active substances, adverse events | No | A data coding dictionary (e.g. MedDRA and WHO drug) has not been incorporated in the EDC system for the glaucoma registry yet. |
| 5 | 5) Quality management [1] Consistency:  The formats and definitions of the data entered in the registry are consistent over time and across different registries | Yes | Data consistency across sites and over time is ensured by use of a standardized data input format used in the EDC system and controlled by the EDC operating manual and CRF data entry manual. Consistency across different registries is not applicable for the glaucoma registry. |
| 6 | [2] Completeness:  Complete information on all eligible patients is recorded, with verification of missing data to keep these at a minimum. | Yes | The monitoring plan pre-specified regular on-site monitoring by a designated site monitor at least every 3 months to perform SDV for all key data fields. Remote monitoring by data manager was planned to monitor compliance to the protocol through the EDC system at the frequency of at least every 3 months. Missing or out of range values are checked automatically in the EDC system and reviewed in on-site and remote (central) monitoring at levels predefined in the manuals. All relevant queries should be addressed. Ensuring that there are no missing data in the critical data is predefined in the monitoring plan. |
| 7 | [3] Accuracy:  The data available in the registry are a correct representation of patient data. | Yes | Data transcription and entry of study data from verifiable source documents, including EHR to the EDC system, is conducted manually by designated trained users. The process of data entry from source documents to CRF is standardized in the EDC operating manual and CRF data entry manual. Missing or out of range values are checked automatically in the EDC system and reviewed in on-site and remote (central) monitoring at the level predefined by the manuals. All relevant queries should be addressed. Ensuring that there are no missing data in the critical data is predefined in the monitoring plan. |
| 8 | [4] Timeliness:  There is a timely recording and reporting of data based on their intended use and in compliance with an agreed procedure. | Yes | The protocol stipulates that data are entered in the CRF immediately after each observation or examination of each participant is completed. The timeliness of data entry in the CRF is reviewed in on-site and remote (central) monitoring. |
| 9 | [5] Registry coordinators should provide to local registries and centers harmonized definitions and data elements | - | Not applicable due to the single registry. |
| 10 | [6] SOPs, work instructions, manuals and users’ guide should be developed, distributed, maintained, and updated as necessary | Yes | The quality management system of the registry is established, which systematically consolidates the SOP and relevant operational procedures. |
| 11 | [7] Training sessions on processes should be organized for local registry coordinators, data managers and data custodians as applicable. | Yes | The process of data entry from source documents to CRF is standardized by the EDC operating manual and CRF data entry manual. Data entry users underwent the first EDC user training concerning these manuals at the start-up meeting and were granted training certification before starting the process of data entry. Ad-hoc user training is performed as manuals are revised and new study staff joins. |
| 12 | [8] If legally and technically possible, a linkage system with other national databases to double-check data or extract additional information should be established. | - | Not applicable due to the single registry. |
| 13 | [9] Routine descriptive statistical analyses should be performed at each data upload into the central database to detect missing data, inconsistent data, outliers, and losses to follow-up | Yes | Missing or out of range values are checked automatically in the EDC system and reviewed in on-site and remote (central) monitoring at the levels predefined in the manuals. All relevant queries should be addressed. Ensuring that there are no missing data in the critical data is predefined in the monitoring plan. |
| 14 | [10] Internal or external audit with on-site review of processes and data audit | Yes | Regular audit is planned to ensure the quality of the glaucoma registry. |
| 15 | [11] Registry data can be benchmarked to an external data source such as national electronic health records to compare the distribution of categories of important variables such as age, gender, or prevalence of disease-related drug exposure. | Yes | The EDC system is designed that the registry data can be output to SAS format for analysis. |
| 16 | [12] Indicators of data quality are defined and measured periodically | Yes | Risk indicators (e.g. rate of blank pages or nonresponse to queries) are defined and reviewed in the on-site monitoring and remote (central) monitoring. |
| 17 | [13] Registries may take the opportunity of a regulatory Qualification of Registries by the Committee for Medicinal Products for Human Use (CHMP) | - | Not applicable |
| 18 | [14] Adequate training of data managers and other persons involved in data entry is critical. | Yes | The process of data entry from source documents to CRF is standardized by the EDC operating manual and CRF data entry manual. Data entry users underwent the first EDC user training concerning these manuals at the start-up meeting and were granted training certification before starting the process of data entry. Ad-hoc user training is performed, such as when manuals are revised, and new study staff joins. |
| 19 | [15] Automated data quality checks and visual prompts should be in place at data entry to prevent introduction of erroneous or inconsistent data and trigger source data verification. | Yes | Missing or out of range values are checked automatically in the EDC system and reviewed in on-site and remote (central) monitoring at the level predefined in the manuals. All relevant queries should be addressed. Ensuring that there are no missing data in the critical data is predefined in the monitoring plan. |
| 20 | [16] Standard data quality control reports should be produced at a local level to check for missing, unusual or incorrect data | Yes | Missing, unusual or incorrect data are reported in the monitoring reports for on-site and remote (central) monitoring. |
| 21 | [17] The procedure for data submission to the central registry should include in a first stage a check of the completeness of the data, and provide the sender a missing data report to increase completeness | Yes | The data entry user should conduct self-checking of data to prevent the registration of incorrect data. The process of data entry from source documents to CRF is standardized in the EDC operating manual and CRF data entry manual. Data entry users underwent the first EDC user training concerning these manuals at the start-up meeting and were granted training certification before starting the process of data entry. Ad-hoc user training is performed as manuals are revised and new study staff joins. |
| 22 | 6) Safety analysis | - | Not applicable |
| 23 | 7) Governance:  Most registries and potential users of registry data have a governance model | Yes | The registry organization was established by collaboration between Tohoku University and Keio University. A system owner took full responsibility to supervise the whole project of the registry, and a simple governance structure without establishing the steering committee was applied. A study manager is responsible for conducting the study and managing the quality of the registry. The study manager assigns a data entry operator, imaging data manager, document manager, auditor, monitor, data manager, and CSV manager. Almost all the foreseeable operations of the registry have been operationalized in the form of the SOPs, and outstanding issues and unpredictable changes in the operation were to be discussed on ad hoc basis. |
| 24 | 8) Data ownership and intellectual property | Yes | The protocol of the registry was reviewed and approved by an ethical review board and stipulates the process of informed consent from participants. The registry data is used and shared in accordance with informed consent provided by subjects. |
| 25 | 9) Data sharing | Yes | Registry data is stored in the EDC system so that regulatory authorities can make contact to verify essential information for decision making. |
| 26 | 10) Informed consent | Yes | The protocol of the registry was reviewed and approved by an ethical review board and stipulates the process of informed consent from participants. |
| 27 | 11) Data security | Yes | A data security system for Medrio EDC has been established. |

| **IMDRF: Tools for Assessing the Usability of Registries in Support of Regulatory Decision-Making** | | | |
| --- | --- | --- | --- |
| **No.** | **Recommendation in the guidance** | **Does the Glaucoma registry meet the recommendation?** | |
| 1 | 1) Governance Governance structure and process | Yes | The registry organization was established by collaboration between Tohoku University and Keio University. A system owner took full responsibility to supervise the whole project of the registry, and a simple governance structure without establishing the steering committee was applied. A study manager is responsible for conducting the study and managing the quality of the registry. A study manager assigns a data entry operator, imaging data manager, document manager, auditor, monitor, data manager, and CSV manager. Almost all the foreseeable operations of the registry have been operationalized in the form of the SOPs, and outstanding issues and unpredictable changes in the operation were to be discussed on ad hoc basis. |
| 2 | 2) Quality management system [1] Legal requirements for data collection/handling, policy on COI | Yes | Handling of personal information is specified in the protocol and complied with the ethical guidelines for medical and health research involving human subjects. |
| 3 | [2] Information on Patient Data Protection (e.g. if Exempt from consent, Opt-out, Opt-in) | Yes | Anonymization of personal information is specified in the informed consent document. The protocol of the registry was reviewed and approved by an ethical review board and stipulates the process of informed consent from participants. |
| 4 | [3] Policy on access to data | Yes | The EDC user management manual provided the policy and procedures to control data access for EDC users. The data access policy allowed only the designated EDC users to have access to the data captured in the field of the EDC according to the user’s roles. The procedures for locking and exporting the data to generate a research dataset was stipulated for researchers within the study team. The SOP for provision of the registry data to investigators outside of the research organization has not been filed. |
| 5 | [4] Essential information available for verification by a relevant authority (e.g. competent authority, notified body) | Yes | Registry data is stored in the EDC system so that regulatory authorities can make contact to verify essential information for decision making. |
| 6 | 3) Data gathering  [1] Relevant Variables | Yes | Relevant variables include demographic factors (age, sex, height, weight), medical history, co-morbidities, ophthalmology testing (Intraocular pressure, Fundoscopy, Stereo-fundoscopy, Visual field, LSFG, OCT, Visual acuity, Refractometry, Measurement of corneal thickness, axial length, anterior chamber depth) and VFQ-25 in the registry. |
| 7 | [2] Unambiguous Device Identification | - | Not applicable because this recommendation is specific for medical devices. |
| 8 | [3] Linkability (Registry with other data source) | Yes | The source, type, format and method of data generation from the registry is defined and identified in the data flow diagram before starting data collection. Data stored in the EDC system and imaging data generated in ophthalmology testing are linked using the common registry ID. |
| 9 | [4] Use of controlled vocabularies | No | A data coding dictionary (e.g. MedDRA and WHO drug) has not yet been incorporated in the EDC system for the glaucoma registry. |
| 10 | [5] Use of a nationally or internationally harmonized minimum data model | Yes | Data collection formats and structures are configured in accordance with CDASH and the common entry form equipped in Medrio EDC, except for examinations and diagnostic images specific for ophthalmology. The definitions of the data elements and fields for ophthalmology tests are configurated in reference to existing clinical trials of glaucoma. Data capture is conducted in the EDC system. |
| 11 | 4) Data storage Security protection against hacking, altering, deleting or stealing data | Yes | Access rights to the registry are configured to differ depending on the user’s role (e.g. investigator, monitor, data manager). The method of granting user access rights is specified in the user management procedure manual. A data security system for Medrio EDC has been established. |
| 12 | 5) Methodologies leading to actionable data [1] Conduct of analysis across different types of analysis frameworks | - | Not applicable as of the development phase |
| 13 | [2] Data Interpretation | - | Not applicable as of the development phase |
| 14 | 6) Transparency/Display/Distribution [1] Report; Key elements and frequency of reports | Yes | Protocol synopsis and funding source of the registry are disclosed in the University Hospital Medical Information Network Clinical Trials Registry (UMIN-CTR). |
| 15 | [2] Website and web-reporting | No | A specific website for the glaucoma registry has not been established. |

| **MHLW: Points to consider for Ensuring the Reliability in Utilization of Registry Data for Applications** | | | |
| --- | --- | --- | --- |
| **No.** | **Recommendation in the guidance** | **Does the Glaucoma registry meet the requirement?** | |
| 1 | 1) Governance by registry holders [1] Establishment of operation and management systems | Yes | The registry organization was established by collaboration between Tohoku University and Keio University. A system owner took full responsibility to supervise the whole project of the registry, and a simple governance structure without establishing the steering committee was applied. A study manager is responsible for conducting the study and managing the quality of the registry. The study manager assigns a data entry operator, imaging data manager, document manager, auditor, monitor, data manager, and CSV manager. Almost all the foreseeable operations of the registry have been operationalized in the form of the SOPs, and outstanding issues and unpredictable changes in the operation were to be discussed on ad hoc basis. |
| 2 | [2] Policy on securing transparency | No | A COI policy has not been opened to the public, although it is described in the protocol. A synopsis of the registry (not full protocol) is disclosed in the University Hospital Medical Information Network (UMIN). |
| 3 | [3] Policy on access to registry data | Yes | Access rights to the registry are configured to differ depending on the user’s role (e.g. investigator, monitor, data manager). The method of granting user access rights is specified in the user management procedure manual. |
| 4 | 2) Computerized system [1] Quality management of the computerized system Methods of quality management of the computerized system may vary depending on the purpose of each registry, etc. | Yes | CSV was completed using the V-model method. The glaucoma registry uses Medrio EDC, an EDC system which complies with ERES guidelines to establish the authenticity, readability, and storage of electromagnetic records. |
| 5 | [2] Security of computerized system Registry holders shall specify the overall security of the computerized system to be utilized and implement it in accordance with the procedures. | Yes | A data security system for Medrio EDC has been established. |
| 6 | [3] Backup and recovery of registry data Registry holders shall specify the methods of backup and recovery of registry data and implement them in accordance with the procedures. | Yes | A data backup system for Medrio EDC has been established. |
| 7 | 3) Quality Management of registry data [1] Data collection methods Regardless of data collection route, registry holders shall specify the methods to appropriately collect predetermined survey items and implement data collection in accordance with the procedure. | Yes | The process of data entry from source documents to CRF is standardized in the EDC operating manual and CRF data entry manual. Data entry users underwent the first EDC user training concerning these manuals at the start-up meeting and were granted training certification before starting the process of data entry. Ad-hoc user training is performed, such as when manuals are revised, and new study staff joins. |
| 8 | [2] Handling of collected registry data Registry holders shall predetermine procedures and handle the collected data in accordance with the procedures. | No | Although data cleaning and data fix methods for the registry are specified in the data management manual, a data coding dictionary (e.g. MedDRA and WHO drug) has not been incorporated in the EDC system for the glaucoma registry yet. |
| 9 | [3] Monitoring If registry holders implement monitoring, registry holders shall predetermine procedures for monitoring, and implement monitoring in accordance with the procedures. | Yes | The monitoring plan pre-specified regular on-site monitoring by a designated site monitor at least every 3 months to perform SDV for all key data fields. Remote monitoring by data manager was planned to monitor compliance to the protocol through the EDC system at the frequency of at least every 3 months. |
| 10 | [4] Quality management for data migration from hospital information system, etc. to a computerized system | - | Not applicable |
| 11 | 4) Quality Assurance for Registry Registry holders shall confirm that they have assured the following matters according to the original purpose of the registry and the quality of registry data. - governance by the registry holder is maintained - quality of registry data is managed | Yes | A quality management system for the glaucoma registry was constructed by establishment of governance, CSV, and implementation of risk assessment and control. Regular audit is planned to ensure the quality of the glaucoma registry. |
| 12 | 5) Data Extraction and Dataset Preparation When an applicant utilizes registry data as application data/documents, the statistical analysis of registry data can be classified into the following two cases. - Registry holders perform the operations from extracting data to execution of the statistical analysis. - The applicant receives a dataset from the registry holder and conducts the statistical analysis. | Yes | Registry data can be output to SAS format for data analysis. A detailed analysis plan will be stipulated in the statistical analysis plan in accordance with the purpose of the secondary use of data. |
| 13 | 6) Consideration for protection of personal information Protection of personal information should be considered regardless of the method used for data quality management. | Yes | The protocol of the registry was reviewed and approved by an ethical review board, and stipulates the process of informed consent from participants. Handling of personal information and anonymization of personal information are specified in the registry protocol. |

Abbreviations: CDASH, Clinical Data Acquisition Standards Harmonization; COI, conflict of interest; CRF, case report form; CSV, computerized system validation; EDC, electronic data capture; EHR, electronic health record; LSFG, laser speckle flowgraphy; OCT, optical coherence tomography; SDV, source data verification; SOP, standard operating procedure; VFQ, Visual Function Questionnaire
