# Supplementary material for: A quality management system aiming to ensure regulatory-grade data quality in a glaucoma registry
Source: PLoS One. 2023 Jun 2;18(6):e0286669. doi: 10.1371/journal.pone.0286669 (PMC10237471; doi:10.1371/journal.pone.0286669)
Supplement: S1 File — (DOCX) [file pone.0286669.s003.docx]

**S1 File. Protocol synopsis: the glaucoma patient registry**

1. Purpose

To construct a glaucoma disease registry that prospectively collects long-term follow-up data of Japanese glaucoma patients, and serves as a potential historical control for comparison with glaucoma patients treated with an investigational drug in future single-arm clinical trials.

1. Target patients

Japanese glaucoma patients who are diagnosed with glaucoma, including primary open angle glaucoma (POAG), primary angle closure glaucoma (PACG), normal tension glaucoma (NTG), preperimetric glaucoma (PPG), ocular hypertension, pseudoexfoliative glaucoma (PEG) and secondary glaucoma

1. Duration of registration

5 years

1. Target sample size

3000 patients

1. Inclusion criteria
2. Patients aged 20 years of age and above at the time of informed consent
3. Patients who give written informed consent to join the registry
4. Patients who are diagnosed with glaucoma, including POAG, PACG, NTG, PPG, ocular hypertension, PEG and secondary glaucoma
5. Exclusion criteria
6. Patients who receive unapproved or off-label treatment in a clinical trial (sponsor-initiated clinical trial, investigator-initiated clinical trial or expanded access program), advanced medical care or specific clinical research
7. Patients with serious systemic diseases (cardiovascular disorder, hepatic disorder, renal disorder, endocrine system disease) who are judged to be inappropriate for joining the registry by the investigator
8. Patients who are judged to be inappropriate for joining the registry by the investigator
9. Examination and observation schedule

| **Examination/observation item** | **Method** | **Period (window)** | | |
| --- | --- | --- | --- | --- |
|  |  | **Enrollment** | **Every 4 months**  **(+/- 1 month)** | **Every 12 months**  **(+/- 1 month)** |
| Patient background | Interview | X |  |  |
| Height and weight | Measurements of height and weight | X |  |  |
| Pulse rate and blood pressure | Measurements of pulse rate, SBP, and DBP |  | X |  |
| Intraocular pressure | Measurements of intraocular pressure by Goldmann applanation tonometer |  | X |  |
| Fundoscopy | Measurement of abnormal findings in fundus by Swept-source OCT (DRI OCT Triton, Topcon Corp., Tokyo, Japan) |  | X |  |
| Stereo fundoscopy | Measurement of abnormal findings in fundus and assessment of optic disc shape by stereo fundus camera (nonmyd WX, Kowa Company, Ltd., Aichi, Japan) |  | X |  |
| Visual field | Measurement of visual field (MD and TD) by Humphrey visual field analyzer (Carl Zeiss Meditec, Dublin, CA) using the Swedish interactive threshold algorithm (SITA)-standard strategy of the 24–2 program. |  | X |  |
| Ocular blood flow | Measurement of optic disc blood flow and waveform by LSFG-NAVI device (Softcare Co., Ltd., Fukutsu, Japan) |  | X |  |
| OCT | Measurement of optic disc structure (cpRNFL), macular (mRNFL, mIPL+GCL, GCC), near retina shape and retinal optic nerve vessels (RPC, VD) by swept-source OCT (DRI OCT Triton, Topcon Corp., Tokyo, Japan) |  | X |  |
| Visual acuity | Measurement of best corrected visual acuity (decimal visual acuity) |  | X |  |
| Refractometry | Measurement of spherical power, cylinder frequency and spherical equivalent |  |  | X |
| Measurement of corneal thickness, axial length, anterior chamber depth | Measurement of corneal thickness and anterior chamber depth by anterior-segment OCT (Casia 2, Tomey Corporation, Nagoya, Japan), and axial length by optical biometer (OA-2000, Tomey Corporation, Nagoya, Japan) |  |  | X |
| QOL (VFQ-25) | Self-administration of NEI VFQ-25 questionnaire |  |  | X |
| Oxidative stress | Measurement of dROM, BAP by free radical analyzer (Free Carpe Diem, Wismerll Co., Ltd., Tokyo, Japan), and skin AGE by AGE Reader (DiagnOptics BV, Groningen, The Netherlands) and AGE Reader software V.2.3 (SAS Institute, Cary, North Carolina, USA) |  |  | X |

Abbreviations: AGE, advanced glycoxidation end products; BAP, biological antioxidant potential; DBP, diastolic blood pressure; GCC, ganglion cell complex; GCL, ganglion cell layer; IPL, inner plexiform layer; LSFG, laser speckle flowgraphy; MD, mean deviations; NEI VFQ-25, The 25-item National Eye Institute Visual Function Questionnaire; OCT, optical coherence tomography; RNFL, retinal nerve fiber layer; ROM, reactive oxygen metabolites; RPC, radial peripapillary capillary; SBP, systolic blood pressure; TD, total deviations; VD, vessel density
