# Supplementary material for: A quality management system aiming to ensure regulatory-grade data quality in a glaucoma registry
Source: PLoS One. 2023 Jun 2;18(6):e0286669. doi: 10.1371/journal.pone.0286669 (PMC10237471; doi:10.1371/journal.pone.0286669)
Supplement: Attachment — Submitted filename: Response to Reviewers_20230509.docx [file pone.0286669.s004.docx]

**RESPONSE TO REVIEWER COMMENTS**

**Reviewer #1:**

Thank you for submitting this manuscript which would be a useful addition to the literature that is accumulating on the quality assessment of disease registries that collect real world data. The focus of the work over here is to compare the quality of a registry against the criteria set by a number of international bodies for a registry to function as a suitable data resource for regulatory purposes. The authors show that their glaucoma registry ticks the majority of criteria and in the process provide a blue print that can be followed by other registries.

The manuscript would be further enhanced if the authors can consider the following points:

1. Can the authors provide further information on the quality assurance process - specifically, how often do they propose to perform source data checks and will those exercises as well as do these exercise need to be performed remotely or physically.

**Response**: Thank you for your suggestion. We clarified the description regarding on-site and remote monitoring in the quality assurance process in the manuscript (p.18, line 367-374) as follows and revised corresponding parts of S2 Table (item #11 and #12 in FDA guideline, item #6 in EMA guideline, and item #9 in MHLW guideline).

To reduce the chance of data errors in the major ophthalmology measurements and to ensure agreement of the data between the source documents and the EDC, the monitoring plan pre-specified regular on-site monitoring by a designated site monitor at least every 3 months to perform source data verification (SDV) for all key data fields, including ocular pressure, visual field, visual acuity, and OCT. Remote monitoring by data manager was planned to monitor compliance to the protocol through the EDC system, especially focusing on errors, missing and inconsistency of the data at the frequency of at least every 3 months.

2. The authors explain that the registry has protocols for data access but it seems that these are for those using the registry. They do not make it clear as to what is the protocol for data access by the wider group of investigators who may or may not be users of the registry. It’s not clear as to what is the data access policy for research.

**Response**: Thank you for your suggestion. We revised the manuscript (p.13, line 275-282) and the S2 Table (item #4 in IMDRF guideline) for clarification as follows.

The standard operating procedure (SOP) of electronic data capture (EDC) user management manual (L1-THO-SOP-005) provided the policy and procedures to control data access for EDC users of the registry. The data access policy allowed only the designated EDC users to have access to the data captured in the field of the EDC according to the user’s roles. For example, the investigators are able to only edit the data of their own site. The procedures for locking and exporting the data to generate a research dataset was stipulated for investigations and researchers within the study team. We have not filed the SOP for provision of the registry data to investigators outside of the current research organization.

3. On the point of governance, it is unclear as to who is in the steering committee and how is this managed

**Response**: Thank you for this suggestion. We added the following sentence in the manuscript (p.9, line 199-207) and revised corresponding parts of the S2 Table (item #23 in EMA guideline, item #101 in IMDRF guideline and item #1 in MHLW guideline).

Considering a small number of study sites, the system owner took full responsibility to supervise the whole project of the present registry, and we sought a simple governance structure without establishing the steering committee according to the recommendation of the AHRQ guidance. Almost all the foreseeable operations of the registry including change control have already been operationalized in the form of the SOPs and manuals. Outstanding issues and unpredictable changes in the operations and surrounding environment of the registry were to be discussed among the system owner, study manager, data manager and computerized system validation (CSV) manager on ad hoc basis, if applicable.

4. Data harmonisation - it seems that the registry has several uniques fields which are preclude it from data harmonisation; I think this needs some greater discussion; how were these fields created and who provided the guidance. Given that there may be several glaucoma registries in the world, did the investigators investigate the level of consensus amongst experts before embarking on the development of fields.

**Response**: Thank you for this suggestion. We revised the corresponding part of the S2 Table (item #2 in FDA guideline, item #3 in EMA guideline and item #10 in IMDRF guideline) and added the following text in the manuscript for clarification. (p.14, line 308-315)

The functional specification described the data collection formats and structures while maintaining compatibility with the standards set by the Clinical Data Acquisition Standards Harmonization (CDASH), except for examinations and diagnostic images specific for ophthalmology. The CSV manager configured the EDC system according to the functional specification. We referred to the definitions of the data elements and fields for ophthalmology tests used in the clinical trials of glaucoma, considering that the data of the present registry might be used for an external control arm in clinical trials.

5. It is unclear as to whether patients have been involved in advising on the contents

**Response**:　We added the following sentence in the manuscript. (p.10, line 207-209)

An opportunity of patient involvement in planning and advising development of the registry was not afforded while VFQ-25 was adopted as the key patient reported outcome (PRO) measurement.

6. Training sessions - how often have these been planned for users

**Response**: We revised the manuscript (p.18, line 355-360) to clarify training sessions as follows, and revised corresponding parts of the S2 Table (item #1and #14 in FDA guideline, item #11, #18 and #21 in EMA guideline, and item #7 in MHLW guideline).

The first EDC user training was also held at the start-up meeting for data entry operators, investigators and monitors in accordance with the EDC user management manual (SOP L1-THO-SOP-005). Ad-hoc user training is performed when manuals of CRF data entry or EDC operation are revised and when a new study staff joins, and as deemed necessary in regard to the issues found at regular monitoring.

7. I believe that the supplementary tables (esp table 2) are possibly more important than some of the figures which have been included in the main text as they constitute actual results.

**Response**: Thank you for your suggestion. The S2 Table is a huge, but important table containing agreement to the recommendations by the regulatory guidelines, as you pointed. We would like to remain the S2 Table in the supporting information, considering the size of the table and the data volume contained. We have already included the summary of the results in the main text. We really appreciate that you mentioned the importance of the supplemental table.

**Reviewer #2:**

**Response**: Thank you for your thoughtful comments to our manuscript. Please allow us to explain the objectives and goal of this manuscript. The primary objective is to detail the process of construction of a quality management system for the glaucoma registry based on risk-based three component approach. The outcomes of this manuscript is the degree of agreement with regulatory recommendations as quality index to assess the data quality of the registry, but not the number of enrollment. As pointed out by the reviewer #1, we wish that this paper provides a blueprint when a registry with similar regulatory purposes is to be constructed. We would appreciate if you could take the above into consideration.

1. Please find a suitable title, because it is very poorly framed. It appears that the researcher doesn’t know how to encapsulate the entire idea in the title.

**Response**: Thank you for your suggestion. We shortened the length of the title as follows.

A quality management system aiming to ensure regulatory-grade data quality in a glaucoma registry

2. Reduce the overall length if the abstract and I am also not happy with the English. There is a need to improve style of writing. And also club the entire abstract and remove the headings given in the abstract.

**Response**: Thank you for your suggestion. We had applied a structured abstract because this manuscript is categorized in “Research article”. According to your suggestion, we revised the abstract to shorten the overall length and improved style, and removed the heading. Please see the revised manuscript.

3. The first line of the introduction is poorly written….. Disease/patient registries are……….

**Response**: Thank you for your suggestion. We revised the first line of the introduction. Please see the revised manuscript.

4. I am not even happy with the entire structure of introduction. The very important feature of an introduction is the background and the importance is missing.

**Response**: Thank you for your suggestion. We revised and shortened the section of introduction to clarify the background and rationale of this manuscript as follows. Please see the revised manuscript.

- 1^st^ paragraph: the expectation of disease registry for regulatory-use,
- 2^nd^ paragraph: importance of ensuring data quality of disease registry,
- 3^rd^ paragraph: necessity of glaucoma registry to be used for regulatory-use,
- 4^th^ paragraph: purpose of this report.

5. RM is also very week and only focuses on curate clinical test and PRO findings in patients with 128 glaucoma and provide longitudinal data. How the three component approach will help in solving the problem. What is the sample design, the procedure for reaching to the target audience is also not clear.

**Response**: Thank you for your suggestion. The method section described what we planned for and how we configurated the quality management system to achieve the initial goals set for the registry.

This research is not a clinical study intended for hypothesis testing or making an estimation. The purpose of the registry is to collect clinical data and patient reported outcome (PRO) of the patients with glaucoma to describe the natural course of glaucoma. We see that quality management of the registry data be an integral part of its regulatory use including new drug application (NDA) and post-marketing safety. Therefore, we proposed the three component approach which would be helpful for achieving fit-for-purpose quality of the registry data as suggested by several regulatory guidelines. The background of necessity of the registry data with high quality and the reference guidelines to achieve fit-for-purpose quality of the data have been summarized in the introduction section of the manuscript.

As for sampling and enrollment of patients with glaucoma, we added the following sentence in the manuscript (p.12, line 257-261) for clarification based on the existing S2 Table.

The designated investigators recruited the glaucoma patients in the routine care setting at the study sites. Minimal exclusion criteria were set to allow consecutive patient enrollment in the registry and the investigators registered the eligible patients after they had provided a written informed consent prior to their entry to the registry.

6. Analysis, Findings conclusions are the weaker sections of this research work.

**Response**: Thank you for your suggestion. As we mentioned in the above response to comment #5, the purpose of this manuscript is to describe how we have constructed a patient registry with data quality fit for regulatory use and examine compliance of the registry to the relevant guidelines. The results section described the deliverables in developing the registry quality management system (QMS), including documents and processes of the QMS, according to the plan described in the Method section and how much extent we achieved the intended goals of the registry through the predetermined processes for developing the QMS by verifying its compliance with the recommendations made in the multiple regulatory guidelines. We believe that the above approach taken is suitable to meet the objective in this manuscript.

7. Kindly rewrite the entire paper

**Response**: Taking above reviewer’s suggestions into consideration, we refined the entire paper. Please see the revised manuscript.
